# Supplementary material for: Predicted meta-omics: A potential solution to multi-omics data scarcity in microbiome studies
Source: PLoS One. 2026 Apr 10;21(4):e0345919. doi: 10.1371/journal.pone.0345919 (PMC13068337; doi:10.1371/journal.pone.0345919)
Supplement: S1 Fig — (A) Spearman’s rank correlations obtained by training MelonnPan [16] using our processed data (x-axis) and the data processed by the authors (y-axis). Correlations were computed during training across 10 folds of cross-validation. We include two feature filtering alternatives: less restrictive (left) and more restrictive (right). The original dataset was published by [12] (see also S1 Table). (B) On the left, a confusion matrix for the result highlighted in sub-figure (C). On the right, a confusion matrix taken from Fig 6 in the study published by [12]. (C) Performance of random forest classifiers for three different classification tasks, corresponding to the datasets [12,22,23] in S1 Table. Top mBx features were determined by MelonnPan during cross-validation, with a correlation cut-off equal to 0.3. Abbreviations: Crohn’s disease (CD), ulcerative colitis (UC), healthy control (HC), end-stage renal disease (ESRD), metagenomics (mGx), metabolomics (mBx). (PDF) [file pone.0345919.s001.pdf]

(A)

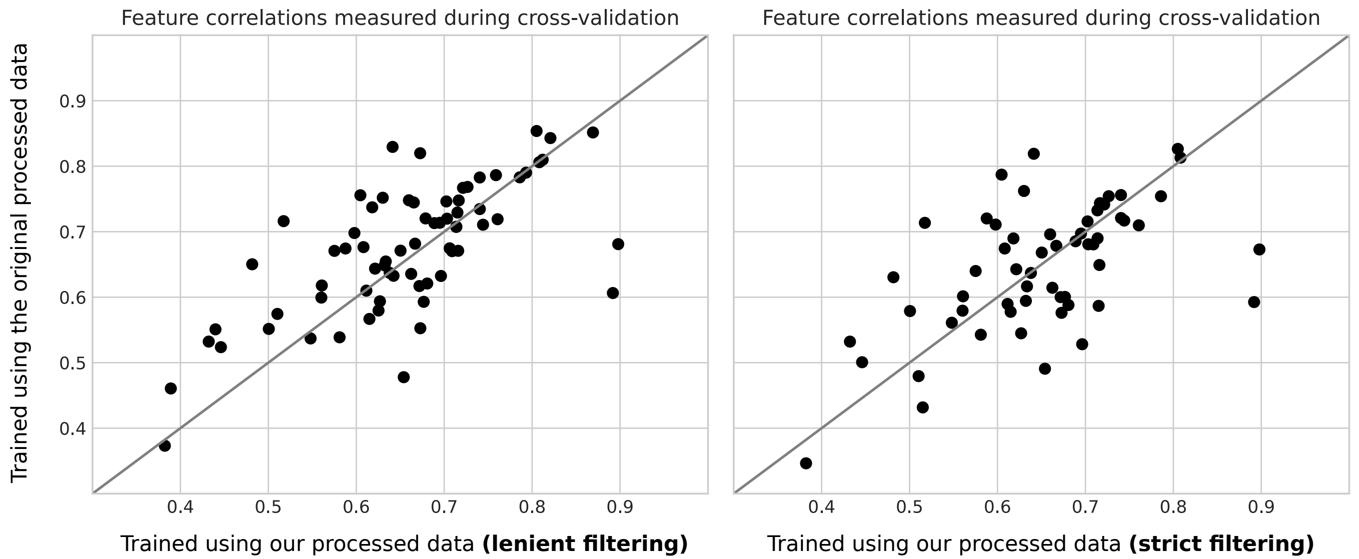

(B)

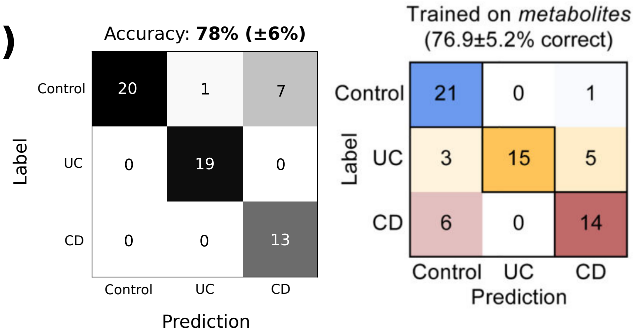

(C)

Lenient filtering

Data	CD, UC and HC	ESRD and HC	Cancer and HC
mGx	56% (±4%)	93% (±3%)	58% (±5%)
mBx	78% (±6%)	99% (±1%)	67% (±3%)
Predicted mBx	63% (±3%)	93% (±6%)	58% (±3%)
Predicted mBx (top features)	62% (±2%)	92% (±5%)	57% (±3%)

Strict fltering

Data	CD, UC and HC	ESRD and HC	Cancer and HC
mGx	58% (±4%)	94% (±4%)	54% (±4%)
mBx	71% (±3%)	86% (±5%)	63% (±3%)
Predicted mBx	58% (±5%)	88% (±6%)	57% (±5%)
Predicted mBx (top features)	58% (±5%)	88% (±6%)	57 (±5%)
