# Supplementary figures and images for: Predicted meta-omics: A potential solution to multi-omics data scarcity in microbiome studies
Source: PLoS One. 2026 Apr 10;21(4):e0345919. doi: 10.1371/journal.pone.0345919 (PMC13068337; doi:10.1371/journal.pone.0345919)

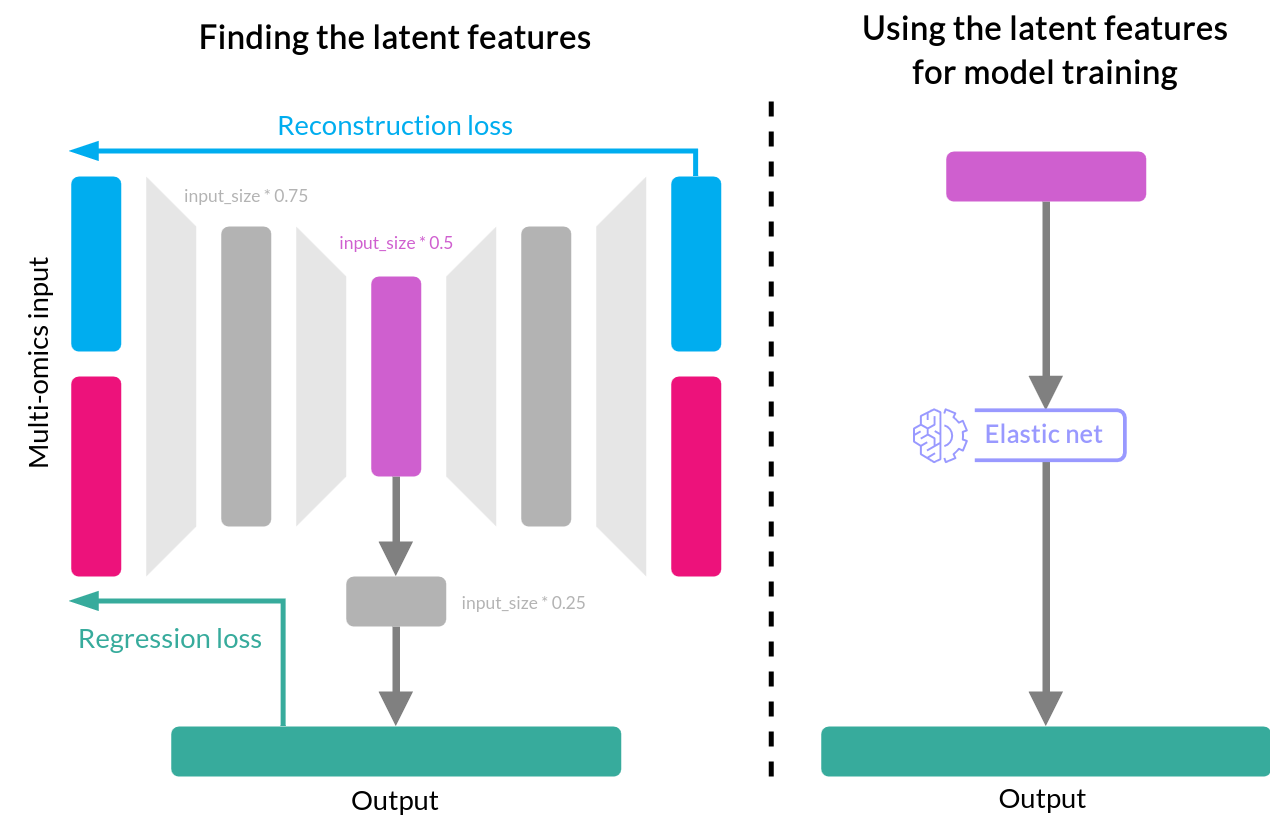

Supplement: S2 Fig — Training a multi-omics autoencoder (S3 Note) with a combined loss, followed by training an elastic net model (MelonnPan [16]) on the latent features. (PNG) [file pone.0345919.s002.png]

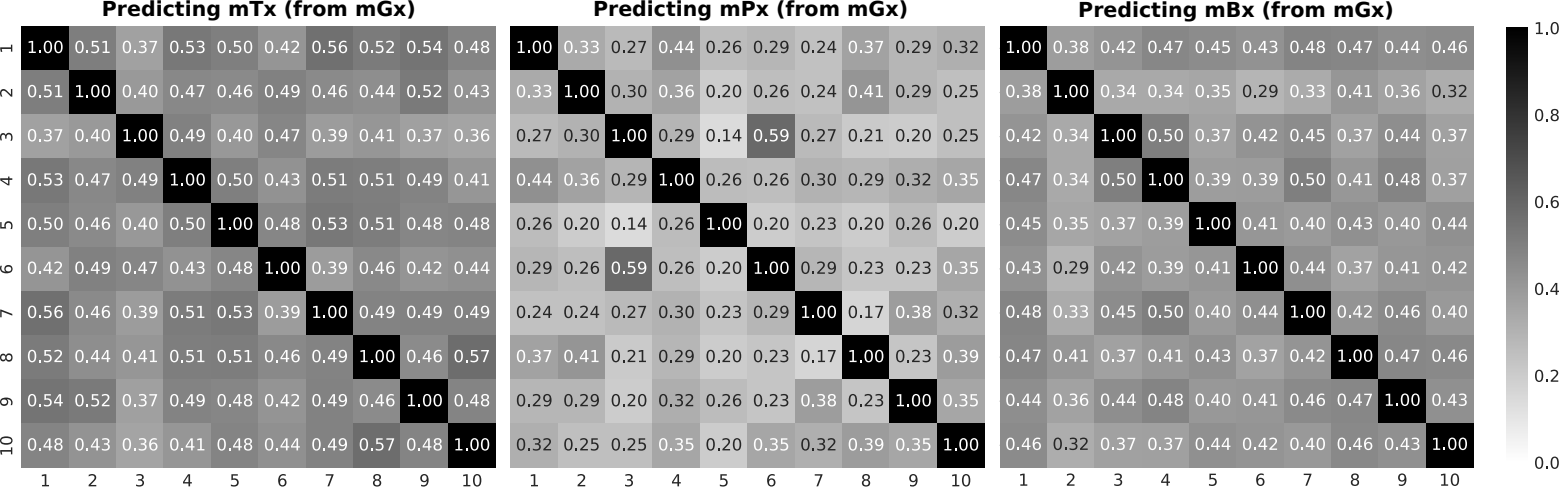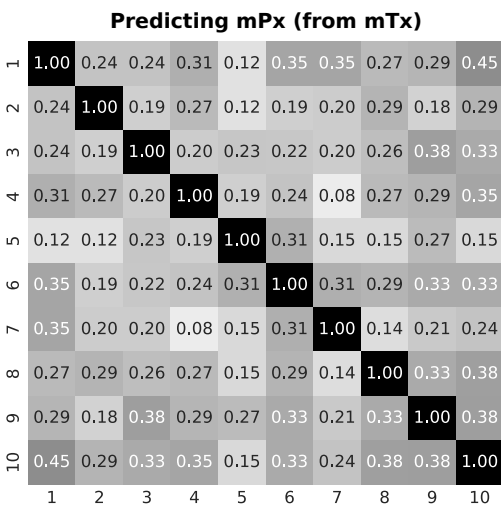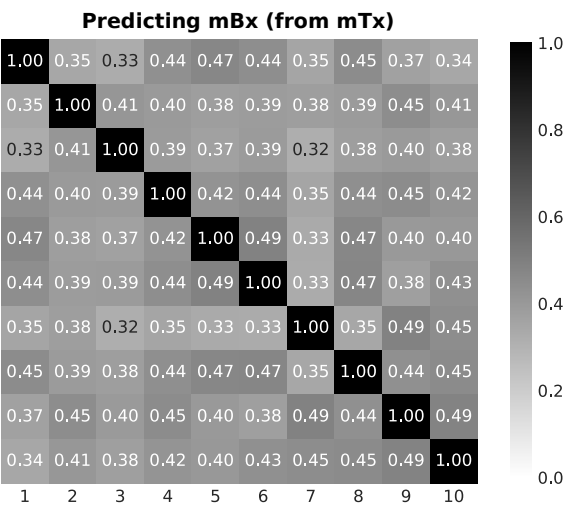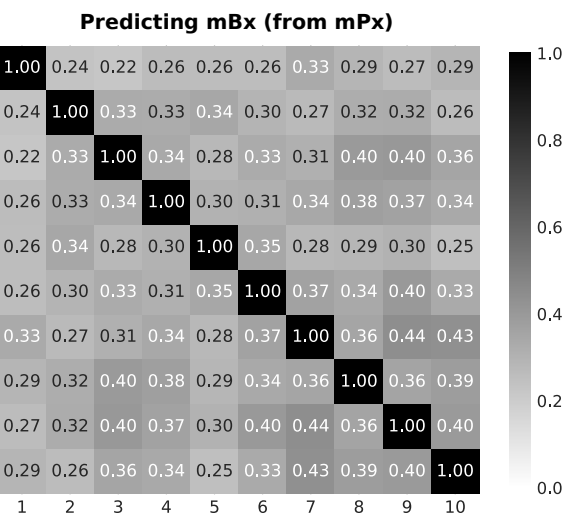

Supplement: S3 Fig — Predictions were generated with MelonnPan [16]. (PDF) [file pone.0345919.s003.pdf]

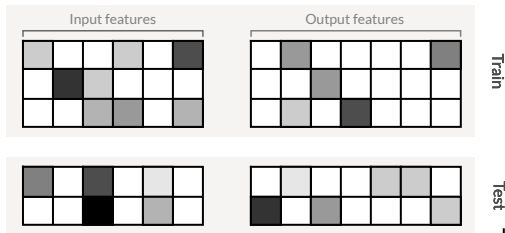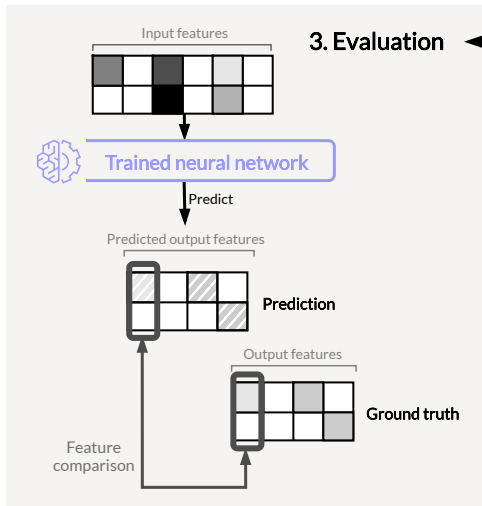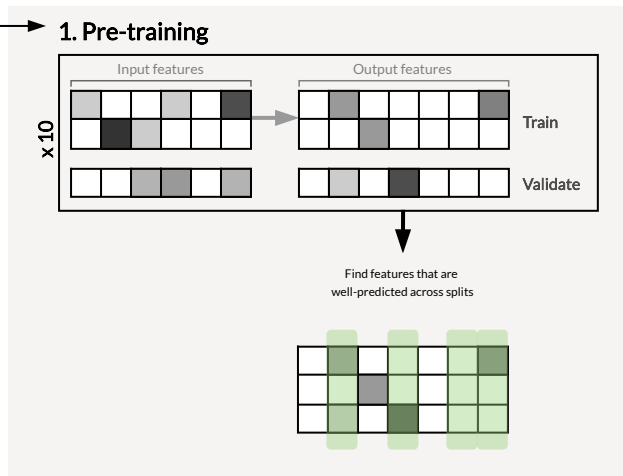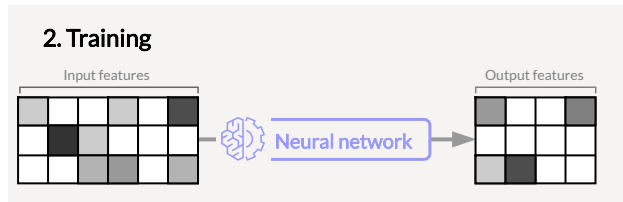

Supplement: S4 Fig — Selected features are subsequently used to train a neural network, as the one described in S3 Note. (PDF) [file pone.0345919.s004.pdf]

Predicting mTx

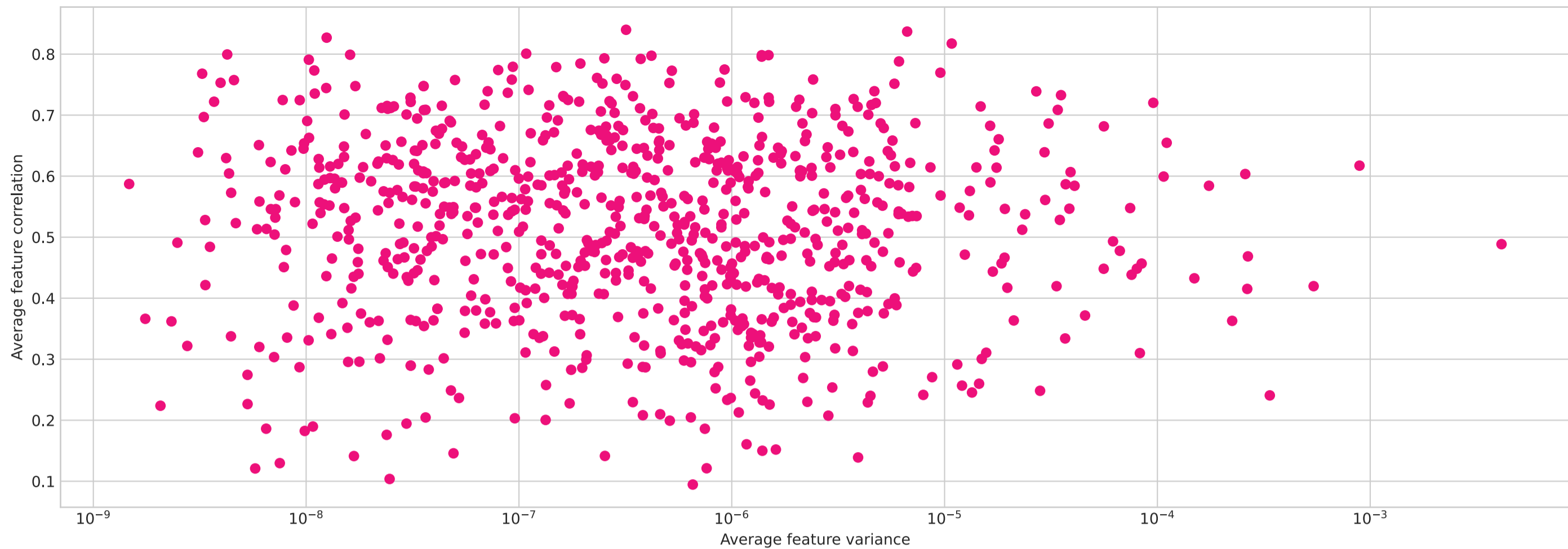

Predicting mPx

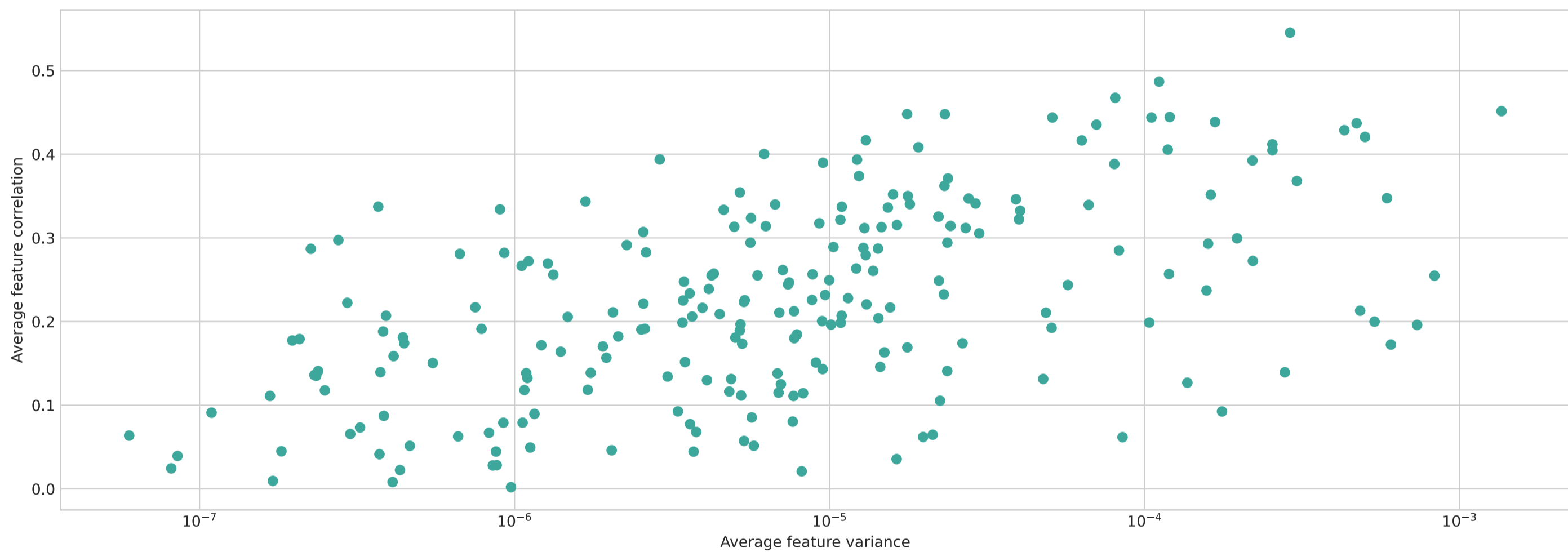

Predicting mBx

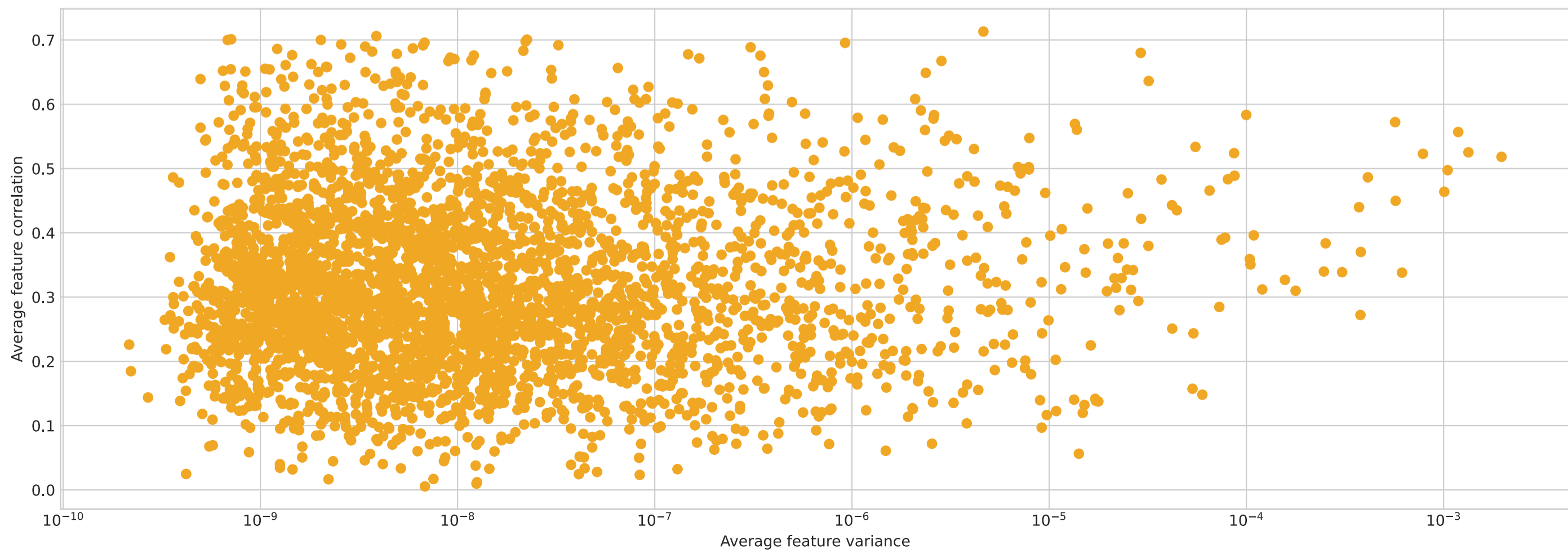

Supplement: S5 Fig — Correlation was computed between predicted features and the ground-truth. Variance and correlation were both computed on test sets, and averaged across dataset partitions and single- and multi-omics input types. (PDF) [file pone.0345919.s005.pdf]
