# Supplementary material for: Predicted meta-omics: A potential solution to multi-omics data scarcity in microbiome studies
Source: PLoS One. 2026 Apr 10;21(4):e0345919. doi: 10.1371/journal.pone.0345919 (PMC13068337; doi:10.1371/journal.pone.0345919)
Supplement: S1 Note — (PDF) [file pone.0345919.s006.pdf]

S1. Computational resources

We ran all experiments on our high-performance computing (HPC) cluster [1] with heterogeneous nodes. Training times ranged from minutes to several days depending on model complexity. Due to variable resource allocation, exact hardware specifications and runtimes are not reported; however, all analyses can be reproduced using standard multi-core CPUs.

References

1. Delft AI Cluster (DAIC). The Delft AI Cluster (DAIC), RRID:SCR_025091. 2024. DOI: 10.4233/rrid:scr_025091. Available from: https://doc.daic.tudelft.nl/
