# Supplementary material for: Predicted meta-omics: A potential solution to multi-omics data scarcity in microbiome studies
Source: PLoS One. 2026 Apr 10;21(4):e0345919. doi: 10.1371/journal.pone.0345919 (PMC13068337; doi:10.1371/journal.pone.0345919)
Supplement: S2 Note — (PDF) [file pone.0345919.s007.pdf]

# S2. Commands for model training

```
# MelonnPan
# Training
Rscript train_metabolites.R -i [path_to_input_data] -g [path_to_output_data] -p 20 -o [path_to_output_folder]
# Making predictions with the re-trained model
Rscript predict_metabolites.R -w [path_to_trained_weights] -i [path_to_input_test_data] -o [path_to_output_folder]


# SparseNED
python3 main_cv_1dir.py --model BiomeAESnip --sparse 0.06 --learning_rate 0.01 --batch_size 20 --latent_size 70 --activation "tanh_tanh" --data_type [dataset_name] --data_root [path_to_data_folder] --nonneg_weight --normalize_input

# MiMeNet
python3 MiMeNet_train.py -micro [path_to_input_data] -metab [path_to_output_data] -micro_norm None -metab_norm None -net_params None -external_micro + [path_to_input_test_data] -external_metab [path_to_output_test_data] -num_background 10 -num_run 5 -num_cv 5
```

**Listing 1:** Commands used to run "metagenomics-to-metabolomics" tools: MelonnPan [1], SparseNED[2] and MiMeNet [3].

## References


1. Mallick H, Franzosa EA, McIver LJ, Banerjee S, Sirota-Madi A, Kostic AD, Clish CB, Vlamakis H, Xavier RJ, and Huttenhower C. Predictive metabolomic profiling of microbial communities using amplicon or metagenomic sequences. Nature Communications. 2019 Jul 17; 10:3136. Available from: https://doi.org/10.1038/s41467-019-10927-1

2. Le V, Quinn TP, Tran T, and Venkatesh S. Deep in the Bowel: Highly Interpretable Neural Encoder-Decoder Networks Predict Gut Metabolites from Gut Microbiome. BMC Genomics. 2020 Jul 20; 21:256. Available from: https://doi.org/10.1186/s12864-020-6652-7

3. Reiman D, Layden BT, and Dai Y. MiMeNet: Exploring microbiome-metabolome relationships using neural networks. PLOS Computational Biology. 2021 May 17; 17:e1009021. Available from: https://doi.org/10.1371/journal.pcbi.1009021
