# Supplementary material for: Predicted meta-omics: A potential solution to multi-omics data scarcity in microbiome studies
Source: PLoS One. 2026 Apr 10;21(4):e0345919. doi: 10.1371/journal.pone.0345919 (PMC13068337; doi:10.1371/journal.pone.0345919)
Supplement: S3 Note — (PDF) [file pone.0345919.s008.pdf]

S3. Supplementary methods

Deep fully-connected neural network

We trained 36 deep neural network (Deep NN) architectures, with various numbers of hidden layers, different data augmentation factors and loss functions. In the end, the architecture that was used throughout our experiments included 3 fully connected hidden layers and a loss function including equal proportions of Pearson's correlation and the mean-squared error (MSE). Additionally, training was performed without any data augmentation. For a comprehensive benchmark of different architectures and augmentation factors, see Supplementary Table S6.

Data augmentation

We augmented the paired cross-omics datasets using an approach inspired by the Aitchison mixup described by Gordon-Rodriguez, Quinn, and Cunningham [1]. However, one important distinction is that the authors describe augmentation of compositional data in the simplex, before transformations are applied, but we apply augmentation on the transformed data. Data was transformed using the quantile transformation.

Let x_i and $x_j \in \mathbb{R}^D$ be two input samples, with corresponding output samples y_i and $y_j \in \mathbb{R}^M$. We construct augmented data points x' and y' using a linear combination:

$$x' = \lambda \cdot x_i + (1 - \lambda) \cdot x_j,$$

$$y' = \lambda \cdot y_i + (1 - \lambda) \cdot y_j,$$

where $\lambda \in [0, 1]$ and $i, j \in \{1, ..., N\}$. To generate multiple data points, i and j are chosen randomly, and λ is sampled from a uniform distribution.

Architecture

Layer dimensions were chosen based on input size. To that end, we constructed architectures of the form: $\texttt{input\_size} - \lfloor 1.25 \cdot \texttt{input\_size} \rfloor - ... - \lfloor 1.25 \cdot \texttt{input\_size} \rfloor - \lfloor 2.5 \cdot \texttt{input\_size} \rfloor - \texttt{output\_size}$. Layer norm and ReLU were applied after each layer, excluding the output layer.

Loss function

We defined a loss function based on a combination of Pearson's correlation and the mean squared error, between the ground truth $Y \in \mathbb{R}^{N \times M}$ and the prediction $\hat{Y} \in \mathbb{R}^{N \times M}$:

$$L(Y, \hat{Y}) = \alpha_{MSE} \cdot MSE(Y, \hat{Y}) + \alpha_{corr} \cdot (1 - \rho(Y, \hat{Y})), \quad (1)$$

where $\rho(Y, \hat{Y})$ represents the average Pearson correlation coefficient between ground-truth and predicted features, $MSE(Y, \hat{Y})$ is the mean squared error between the ground truth and the prediction, and α_{MSE} and $\alpha_{corr} \in [0, 1]$.

To compute the mean squared error, we used `torch`'s (2.1.2.post300) MSE loss, from the `nn.functional` module, with "mean" reduction. To determine Pearson's correlation coefficient, we applied the CosineEmbeddingLoss from the same module. Prior to this, each feature was centered around its mean, and the data batch was transposed.

Training procedure

All network models (Supplementary Table S6) were constructed using the Pytorch Lightning API, version 2.2.1, with a random seed equal to 42. Training and validation sets were split based on study participants, using a shuffled split, with a random seed equal to 42. We used a batch size of 16 and the Adam optimizer, with a learning rate equal to `1e-4`, a patience of 3 for early stopping, and a maximum of 35 epochs.

Multi-omics autoencoder

As an alternative to naive feature concatenation, we trained an autoencoder model for multi-omics integration. A diagram of the architecture and loss function is included in Supplementary Figure S2. After training the network, we used the latent features to train the best-performing model in our benchmark, MelonnPan [2]. Below we provide details on the network architecture and the loss function used during training.

Architecture

As shown in Supplementary Figure S2, we divided the model architecture into two main parts: the autoencoder and a multi-layer perceptron, which takes as input the latent features of the autoencoder, and then predicts a meta-omics output. The autoencoder was organized using a symmetric architecture, with a hidden layer of dimension $\lfloor 0.75 \cdot \texttt{input\_size} \rfloor$, and a latent space of dimension $\lfloor 0.5 \cdot \texttt{input\_size} \rfloor$. The multi-layer perceptron included one hidden layer, of dimension $\lfloor 0.25 \cdot \texttt{input\_size} \rfloor$. Following a similar approach as described previously (Supplementary Section), we applied layer norm and a ReLU activation after each layer, excluding the output layers.

Loss function

We constructed an architecture to enable learning of embeddings for the task of cross-omics prediction. To that end, we trained an autoencoder with a combined loss, integrating the reconstruction loss with a regression loss. This was

inspired by the approach described by Hira et al. [3], who jointly trained a variational autoencoder and classifier for ovarian cancer, using a combined loss.

Let $X \in \mathbb{R}^{N \times D}$ be the input multi-meta-omics feature matrix, and let $Y \in \mathbb{R}^{N \times M}$ be the output meta-omics feature matrix. In addition, let $\hat{X} \in \mathbb{R}^{N \times D}$ be the prediction produced by the autoencoder, and let $\hat{Y} \in \mathbb{R}^{N \times M}$ be the prediction produced by the multi-layer perceptron. We computed the following loss:

$$L(X, \hat{X}, Y, \hat{Y}) = L(X, \hat{X}) + L(Y, \hat{Y}), \tag{2}$$

where $L(X, \hat{X})$ and $L(Y, \hat{Y})$ are defined as in equation 1.

Training procedure

We followed the same procedure as described in Supplementary Section , with the exception that the maximum number of epochs was set to 50.

Feature selection

We designed a pre-training step to select a small set features to be used later during model training (Supplementary Figure S4). To that end, we split each training set into 10 training/validation partitions, and trained a random forest regressors on each partition. Feature correlations were subsequently calculated on the validation set, and each feature was assigned a score, equal to the mean across validation sets. Based on these scores, we retained a fraction of features to be later used for cross-omics model training.

References

1. Gordon-Rodriguez E, Quinn T, and Cunningham JP. Data augmentation for compositional data: Advancing predictive models of the microbiome. Advances in Neural Information Processing Systems 2022; 35:20551–65

2. Mallick H, Franzosa EA, McIver LJ, Banerjee S, Sirota-Madi A, Kostic AD, Clish CB, Vlamakis H, Xavier RJ, and Huttenhower C. Predictive metabolomic profiling of microbial communities using amplicon or metagenomic sequences. Nature Communications. 2019 Jul 17; 10:3136. Available from: https://doi.org/10.1038/s41467-019-10927-1

3. Hira MT, Razzaque MA, Angione C, Scrivens J, Sawan S, and Sarker M. Integrated multi-omics analysis of ovarian cancer using variational autoencoders. Scientific Reports. 2021 Mar 18; 11:6265. Available from: https://doi.org/10.1038/s41598-021-85285-4
