# Supplementary material for: Predicted meta-omics: A potential solution to multi-omics data scarcity in microbiome studies
Source: PLoS One. 2026 Apr 10;21(4):e0345919. doi: 10.1371/journal.pone.0345919 (PMC13068337; doi:10.1371/journal.pone.0345919)
Supplement: Attachment — Submitted filename: Response to Reviewers.pdf [file pone.0345919.s023.pdf]

Dear Editor and Reviewers,

We would like to thank you for taking the time to consider our work. We revised the manuscript in line with your suggestions, and we believe that this improved its clarity and research depth.

We provided answers to the reviewers' comments in the following pages of this letter. For the comments raised by Reviewer 1, we included these answers in pages 2-3. We responded to the comments raised by Reviewer 2 in pages 4-6.

We amended our Funding statement:

> ***Funding***
>
> *SP is supported wholly/in part by the National Research Foundation of South Africa (Grant Numbers: 120192). The funders had no role in study design, data collection and analysis, decision to publish, or preparation of the manuscript. DCF is affiliated with Hologenomix B.V. The funder provided support in the form of salaries for author DCF, but did not have any additional role in the study design, data collection and analysis, decision to publish, or preparation of the manuscript. The specific roles of these authors are articulated in the 'author contributions' section. There was no additional external funding received for this study.*

We also amended our Competing Interests statement:

> ***Competing interests***
>
> *DCF is affiliated with Hologenomix B.V. This does not alter our adherence to PLOS ONE policies on sharing data and materials.*

All of our answers refer to line numbers included in the manuscript file with track changes (*Revised Manuscript with Track Changes.pdf*), where we highlighted the updates compared to the original manuscript. We also uploaded a clean version of the manuscript (*Manuscript.pdf*).

We appreciate your review of our revised manuscript.

Sincerely,

Thomas Abeel

Reviewer 1

Major Concern Regarding Practical Implications and Conclusions

Reviewer's comment: *The ultimate conclusion provided in the manuscript is currently vague and unsatisfying, particularly regarding the implications for practical microbiome research and disease prediction. Specifically:*
- Clarify Practical Guidance: It is unclear whether researchers are advised to rely on predicted meta-omics values rather than experimental ones, particularly in the context of disease (IBD) classification. Do the authors suggest that predicted meta-omics data can genuinely substitute experimental multi-omics data in clinical or research settings, or is this merely a complementary tool?
- Single-omics vs Multi-omics: The manuscript does not clearly state if researchers would gain more from investing in collecting comprehensive multi-omics datasets or if predicted data from readily available (single-omics) sources provide sufficient insight.

Recommendation:
- Please explicitly state the authors' recommendation. For example, should researchers confidently use predicted values in lieu of experimental multi-omics data for tasks like IBD classification, or is the main utility to augment existing datasets?
- Discuss explicitly under what conditions researchers should prioritize collecting multi-omics datasets versus using predicted meta-omics, considering trade-offs in cost, complexity, accuracy, and reliability.

Addressing these points will significantly enhance the clarity, impact, and practical utility of the manuscript.

Response: We agree that the conclusion right now lacks clarity with regard to the main points raised by the reviewer, namely the reliability and utility of predicted meta-omics. We addressed this in the manuscript as follows. Our Discussion now includes a paragraph (lines **488-509**) in which we clarify that we do not expect predicted data to replace experimental data, but rather to be used more as a tool for hypothesis generation, especially for diagnosis in a clinical setting.

Technical Soundness and Support for Conclusions

Reviewer's comment: *Protein prediction (mPx) remains weak (average correlations ~0.4), which the authors acknowledge, but further discussion of the biological and technical reasons for this limitation would strengthen the conclusions.*

Response: We now discuss metaproteomics predictions separately in lines **439-449**, with a focus on the limitations of mass-spectrometry data and pre-processing approaches.

Reviewer's comment: *The biological interpretation of consistently well-predicted features (e.g., glutamate dehydrogenase and RNA polymerase, page 18) is only briefly touched upon; more in-depth discussion of why these features are robustly predictable would*

improve the manuscript.
Response: We now discuss these predictions further in lines **338-347**. We also added an enrichment analysis of well-predicted features (lines **348-354** and **Supplementary Tables S11 and S12**).

Statistical Analysis

Reviewer's comment: *For classification, the focus is on accuracy. Reporting additional metrics (AUC, precision, recall, F1) would give a fuller picture, particularly given class balance issues acknowledged on page 26.*

Response: We included additional metrics in **Supplementary Table S14** to support the results for IBD classification. We also reference this in our Results, in lines **429-431**.

Reviewer's comment: *Metaproteomics seems to suffer from data availability. Would be nice to know if this could have been overcome by using simulated data.*

Response: We addressed this in lines **503-509** of our Discussion.

Clarity and Writing Quality

Reviewer's comment: *Need to stick to multi-meta-omics (page 18) or multiple meta-omics (page 3). Very inconsistent throughout the manuscript.*

Response: We performed a full manuscript check and replaced all such instances with references to the term multi-meta-omics.

Reviewer's comment: *Figure 3 implies that there should be yellow for the metabolomics but there is no yellow in the chart.*

Response: This was a mistake in the Figure 3 caption and it has been corrected.

Reviewer's comment: *Page 10 starts with a capital A when the previous sentence on page 9 was not complete.*

Response: This was an issue regarding the layout of the manuscript, which we now fixed in the new template.

Reviewer 2

Study Design & Methodology

Reviewer's comment: *Clarify train/test splits, 10-fold cross-validation, and detailed preprocessing (imputation, normalization).*

Response: We agree that this should be more clearly stated for the reader to get a grasp of the steps in our pipeline and their order. To that end, we updated our Methods section to include an overview of the experimental pipeline, in lines **92-101**. We also added a sentence clarifying that the 5-fold cross-validation used to train the IBD classifiers exclusively happens within the training sets for each classifier (lines **209-210**).

Reviewer's comment: *Discuss findings' generalizability, specifically if IBDMDB, CRC, and ESRD datasets adequately represent diverse microbiome contexts.*

Response: We included this in our Discussion section (lines **523-527**).

Reviewer's comment: *Explain how class imbalance was addressed beyond downsampling (e.g., stratified weighting).*

Response: We addressed class imbalance by downsampling to equal class proportions, i.e. stratified downsampling. The classifiers described in Section 3.4 are trained on cross-validation splits that are stratified based on these proportions, i.e. stratified k-fold.

However, in response to this comment, we also reconsidered our evaluation metric for IBD classification. Previously, we were downsampling the test set and reporting the accuracy, but we now opted to leave the test sets as they are and instead report the balanced accuracy (also called weighted accuracy), which considers class proportions in imbalanced datasets.

Figure 5 has been updated to include balanced accuracy, and the underlying data changed. We amended our pipeline with two improvements: (1) the downsampled train/test splits across datasets now include the same samples, rather than just the same number of samples, to ensure fair comparisons; and (2) although seeded, the hyperparameter optimization of random forests was not generating reproducible results due to multi-threading; we now use only one thread for training. We also now downsample based on input-output combination, rather than for a whole output type (i.e. subfigure), to keep variances in the results as low as possible. We also report a dummy classifier per input-output combination.

Although this does not change the main conclusion of our paper regarding predicted data (i.e. that it should be used as a tool to inform experiments and lead to hypothesis generation), we changed our interpretation of these Results to reflect the data in the updated figure (lines **405-431**).

Reviewer's comment: *Provide statistical testing for performance comparisons (e.g.,*

significance of differences between elastic net regression and other models, sparse encoder–decoder, feed-forward neural network, and random forest in predicting meta-omics layers).

Reviewer's comment: *Quantify inter-model variability statistically rather than descriptively.*

Response: We now provide these results in **Supplementary Tables S9 and S13** (to keep the figures legible, we did not modify them to highlight statistical significance). We also updated our Results section to mention these differences (lines **284-285, 412, 425**).

Reviewer's comment: *Consider including a brief statement on computational resource usage (e.g., training runtime, GPU specifications) for reproducibility completeness.*

Response: All experiments were run on a shared cluster, so we cannot report specific resources. GPUs are not required to run these experiments. We now also clarified this in our Supplemental Notes (S1) and referenced it in lines **101-102**.

Reviewer's comment: *Consider a short paragraph on ethical/data governance implications of AI-predicted biological data, aligning with PLOS ONE's "broader impact" expectations.*

Reviewer's comment: *Add a paragraph on the ethical/data governance implications of AI-predicted biological data, per PLOS ONE's "broader impact" guidelines.*

Response: We updated our Discussion addressing concerns regarding AI-predicted data in the context of patient data and diagnosis. (lines **533-536**)

Results & Discussion

Reviewer's comment: *Discuss biological interpretability on how these predictions can inform hypothesis generation, rather than only classification tasks.*

Response: This is now included in lines **494-503** of our Discussion.

Reviewer's comment: *Add enrichment or pathway analysis for the top predicted transcripts, proteins, and metabolites.*

Response: We added enrichment analysis for predicted transcripts and proteins based on enzyme commission (EC) classes (**Supplementary Tables S11 and S12**). We discuss these results in lines **348-354**.

Conclusion

Reviewer's comment: *While limitations like sample size are noted, further discussion on how larger multi-omics repositories could enhance predictive performance would be beneficial.*

Response: We now address this in lines **505-509** of our Discussion.

Reviewer's comment: *Expand on the future applicability of predicted meta-omics (e.g., in personalized medicine or microbial ecology).*

Response: We added a section in our Discussion (lines **495-503**) regarding potential applications of predicted microbiome data.
